# Analytical methods and applications in materials and life sciences

**DOI:** 10.1007/s00216-022-04082-8

**Published:** 2022-04-30

**Authors:** Ute Resch-Genger, Björn Meermann, Matthias Koch, Michael G. Weller

**Affiliations:** Bundesanstalt für Materialforschung und -prüfung (BAM), Richard-Willstätter-Straße 11, 12489 Berlin, Germany

Current trends in materials and life sciences are flanked by the need to push detection limits to single molecules or single cells, enable the characterization of increasingly complex matrices or sophisticated nanostructures, speed up the time of analysis, reduce instrument complexity and costs, and improve the reliability of data. This requires suitable analytical tools such as spectroscopic, separation and imaging techniques, mass spectrometry, and hyphenated techniques as well as sensors and their adaptation to application-specific challenges in the environmental, food, consumer product, health sector, nanotechnology, and bioanalysis. Increasing concerns about health threatening known or emerging pollutants in drinking water, consumer products, and food and about the safety of nanomaterials led to a new awareness of the importance of analytical sciences. Another important driver in this direction is the increasing demand by legislation, particularly in view of the 17 sustainable development goals by the United Nations addressing clean energy, industry, and innovation, sustainable cities, clean water, and responsible consumption and production. In this respect, also the development of analytical methods that enable the characterization of material flows in production processes and support recycling concepts of precious raw materials becomes more and more relevant. In the future, this will provide the basis for greener production in the chemical industry utilizing recycled or sustainable starting materials. This makes analytical chemistry an essential player in terms of the circular economy helping to increase the sustainability of production processes. In the life sciences sector, products based on proteins, such as therapeutic and diagnostic antibodies, increase in importance. These increasingly biotechnologically produced functional biomolecules pose a high level of complexity of matrix and structural features that can be met only by highly advanced methods for separation, characterization, and detection. In addition, metrological traceability and target definition are still significant challenges for the future, particularly in the life sciences. However, innovative reference materials as required for the health and food sector and the characterization of advanced materials can only be developed when suitable analytical protocols are available. The so-called reproducibility crisis in sciences underlines the importance of improved measures of quality control for all kinds of measurements and material characterization. This calls for thorough method validation concepts, suitable reference materials, and regular interlaboratory comparisons of measurements as well as better training of scientists in analytical sciences.

The important contribution of analytical sciences to these developments is highlighted by a broad collection of research papers, trend articles, and critical reviews from these different application fields. Special emphasis is dedicated to often-overlooked quality assurance and reference materials.

